# Crumbs Affects Protein Dynamics In Anterior Regions Of The Developing *Drosophila* Embryo

**DOI:** 10.1371/journal.pone.0058839

**Published:** 2013-03-21

**Authors:** João Firmino, Jean-Yves Tinevez, Elisabeth Knust

**Affiliations:** Max-Planck-Institute of Molecular Cell Biology and Genetics, Dresden, Germany; Baylor Universitym, United States of America

## Abstract

Maintenance of apico-basal polarity is essential for epithelial integrity and requires particular reinforcement during tissue morphogenesis, when cells are reorganised, undergo shape changes and remodel their junctions. It is well established that epithelial integrity during morphogenetic processes depends on the dynamic exchange of adherens junction components, but our knowledge on the dynamics of other proteins and their dynamics during these processes is still limited. The early *Drosophila* embryo is an ideal system to study membrane dynamics during morphogenesis. Here, morphogenetic activities differ along the anterior-posterior axis, with the extending germband showing a high degree of epithelial remodelling. We developed a Fluorescence Recovery After Photobleaching (FRAP) assay with a higher temporal resolution, which allowed the distinction between a fast and a slow component of recovery of membrane proteins during the germband extension stage. We show for the first time that the recovery kinetics of a general membrane marker, SpiderGFP, differs in the anterior and posterior parts of the embryo, which correlates well with the different morphogenetic activities of the respective embryonic regions. Interestingly, absence of *crumbs*, a polarity regulator essential for epithelial integrity in the *Drosophila* embryo, decreases the fast component of SpiderGFP and of the apical marker Stranded at Second-Venus specifically in the anterior region. We suggest that the defects in kinetics observed in *crumbs* mutant embryos are the first signs of tissue instability in this region, explaining the earlier breakdown of the head epidermis in comparison to that of the trunk, and that diffusion in the plasma membrane is affected by the absence of Crumbs.

## Introduction

Epithelia are characterised by a pronounced apico-basal polarity of their cells with the apical side facing the outside and the baso-lateral side facing neighbouring cells and/or a basal lamina. Their cells are closely connected to each other by different types of junction, such as adherens junctions or tight junctions, which guarantee integrity and tightness of these tissues. Epithelia are of crucial importance for shaping the embryo, for example during gastrulation, neurulation or tissue elongation during organogenesis. Several processes contribute to morphogenetic changes of epithelia, such as oriented cell division, changes in cell shape and cell size, remodelling of junctions, reorganisation of the actomyosin cytoskeleton, modification of apical and baso-lateral surface areas and cell intercalation (reviewed in: [1],[2],[3],[4]).

Cell intercalation is the major driving force for tissue and organ elongation and largely depends on convergence and extension movements. It contributes to shaping of embryos and organs and is instrumental for vertebrate axis elongation, tube formation or germband extension in the *Drosophila* embryo, to mention just a few [1],[5],[6],[7]. Germband extension in the fly embryo is an ideal model system to study the genetic and cell biological basis underlying tissue elongation. During elongation, the germband, which develops into the segmented trunk of the larvae, doubles in length along the anterior-posterior axis and narrows along the dorso-ventral axis [8],[9]. The process can be subdivided into the first, rapid phase, which takes about 25 minutes, during which most of elongation occurs and the second, slow phase, covering the following 70 minutes [10],[11]. Several processes contribute to the elongation of the tissue, which differentially affect the anterior and the posterior region of the germband. While tissue elongation in the anterior region mostly depends on cell intercalation [8],[12],[13], taking place as response to mechanical forces exerted by the invaginating mesoderm [14] and anisotropies in cortical tension [15],[16],[17], extension of the posterior region substantially relies on cell divisions oriented along the anterior-posterior axis [18].

During morphogenetic processes, including germband extension, epithelial integrity and polarity are controlled by a number of mechanisms, which are closely interconnected. One of the key regulators of epithelial polarity in the *Drosophila* embryo is the Crumbs complex, which contains the transmembrane protein Crumbs (Crb) and the scaffolding proteins Stardust (Sdt), *D*Lin-7 and *D*PATJ as core components. Other components, such as *D*Par-6, a member of the Par protein group or Yurt, a negative regulator of Crb, can be transiently recruited into the complex (reviewed in [19],[20]). Embryos lacking *crb* function fail to maintain apico-basal polarity in many of their epithelia, which eventually leads to a complete breakdown of tissue integrity, followed by apoptosis [21]. In particular the developing epidermis is strongly affected. Here, an intact Crb complex is essential to position and form the *zonula adherens* (ZA), a belt like structure encircling the apex of the cell [22],[23]. On the other hand, overexpression of Crb can lead to an expansion of the apical membrane domain, both in embryos [24] and photoreceptor cells [25],[26],[27]. These results point to a role of Crb in maintaining the apical membrane, but data demonstrating this role are still missing.

Fluorescence Recovery After Photobleaching (FRAP) is an ideal method for *in vivo* measurements of protein turnover. Using this method, it was recently shown that biosynthetic *D*E-Cadherin turnover was higher at early stages of *Drosophila* embryogenesis, when cells are polarising, compared to polarised epithelia at later stages [28]. Using the same technique, we were interested to find out whether the turnover of general and polarised plasma membrane markers was spatially regulated during germband extension – a stage where cells necessarily need to remodel their plasma membrane and junctions - and whether the polarity regulator Crb plays a role in this process.

## Results

**Figure 1:**
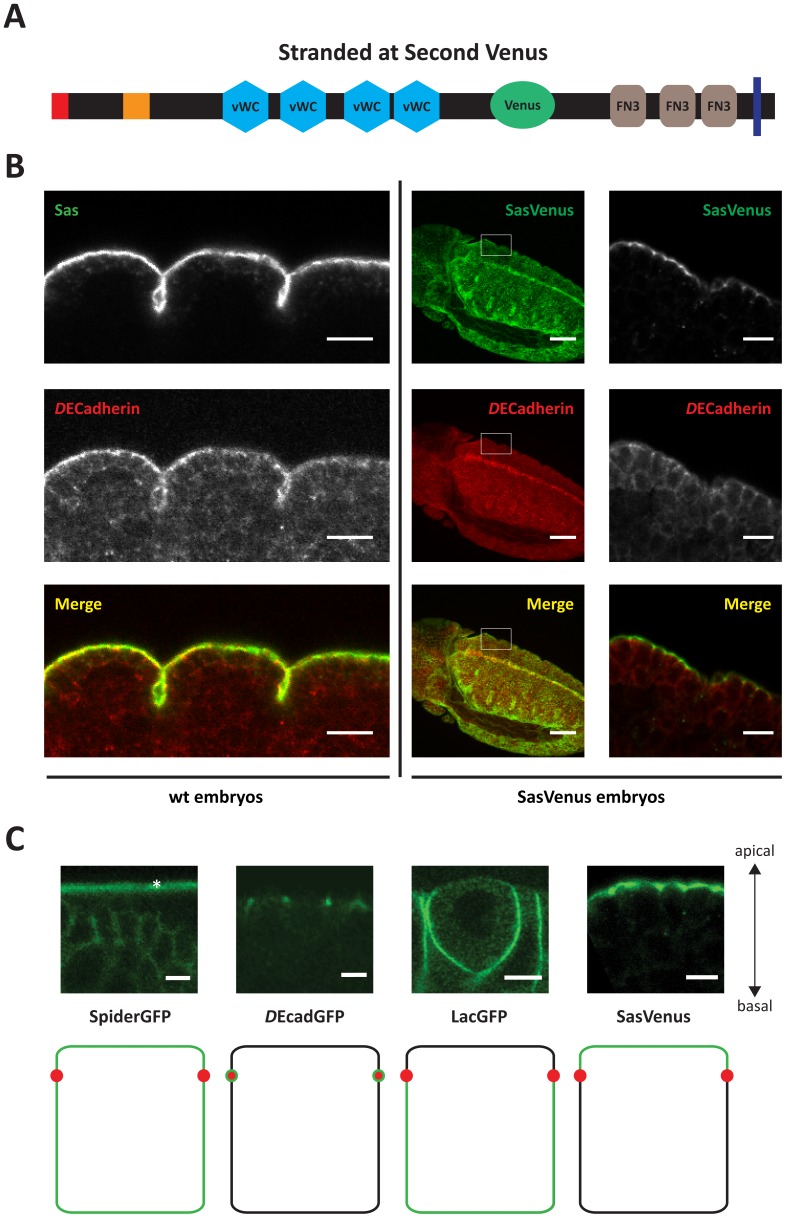
Stranded at Second-Venus and a toolkit of plasma membrane proxies used in this study. **A**) Organisation of Stranded at Second (Sas)-Venus used here. Note that part of the native protein was replaced by the fluorophore (green) (see methods section). vWC: von Willebrand factor type C; FN3: Fibronectin type 3; blue bar: transmembrane domain. **B**) Localisation of the transgene-encoded Sas-Venus resembles the localisation of Sas in wild-type embryos. Left: Sas (top) and *D*E-Cadherin (middle) staining of the epidermis in wild-type embryos of stage 16 (scale bar: 10μm). Right: *D*E-Cadherin expression and Venus fluorescence in a late germ band extension DaGAL4 UAS Sas-Venus embryo (scalebar: 50μm) with corresponding closeups (scalebar: 10μm). **C)** The membrane proxies toolkit – SpiderGFP labels the entire plasma membrane, *D*E-CadherinGFP marks the *zonula adherens*; LachesinGFP labels the basolateral membrane and the transmembrane protein Sas-Venus highlights the apical membrane. The asterisk refers to the vitelline membrane. Scale bar – 5 µm.

To better understand protein dynamics during germband extension in the *Drosophila* embryo, we developed a FRAP assay with a higher temporal resolution. Since an exclusively apical marker was lacking, Stranded at Second (Sas) was fluorescently tagged with Venus (Fig. 1A). Sas is a type I transmembrane protein composed of 1693 amino acids, with four predicted von Willebrand factor type C (vWC) - and three fibronectin 3 (FN3)-domains [29]. Sas is expressed during germband retraction in ectodermally derived tissues, where it is restricted to the apical membrane [24]. A low complexity region of the protein (isoform B; aa 1092–1244) was replaced with Venus, a YFP derived fluorophore with high brightness levels [30],[31] (Fig. 1A) and put under the control of UAS-elements or a tubulin promoter. Flies expressing Sas-Venus from either transgene were viable and fertile and did not exhibit any obvious morphological defects. The localisation of the tagged protein resembled that of the endogenous protein (Fig. 1B), in that both are restricted to the apical plasma membrane, apical to the ZAs. Including this novel apical marker, we could now make use of a complete toolkit of compartment-specific plasma membrane markers: SpiderGFP (also known as Gilgamesh, or casein kinase CK1γ), a protein linked to the membrane via C-terminal palmitoylation, labels the entire plasma membrane [32]; the homophilic cell adhesion molecule *D*E-Cadherin marks the ZA [33]; LachesinGFP, a GPI-linked member of the immunoglobulin superfamily, labels the basolateral membrane similar as the endogenous Lachesin protein [34] and Sas-Venus marks the apical membrane (this study) (Fig. 1C).

**Figure 2:**
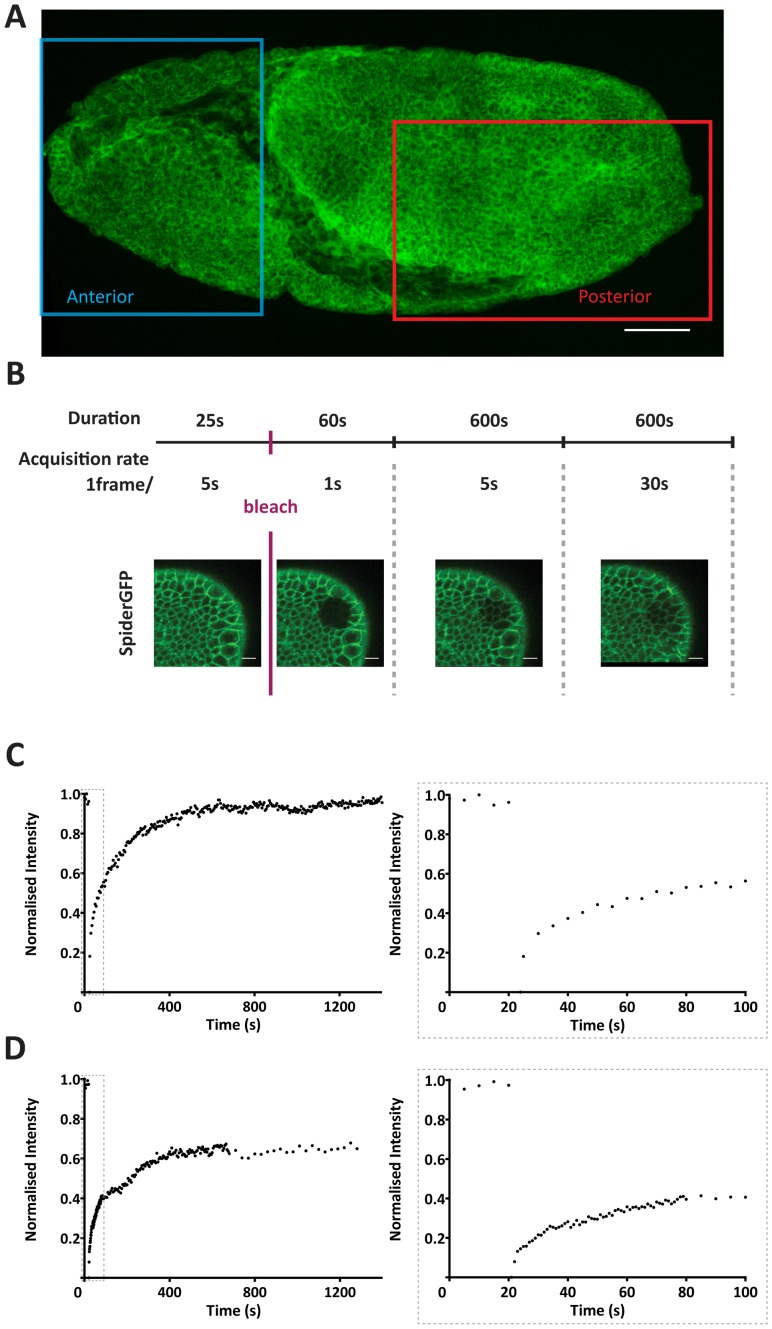
The FRAP assay. **A)** Imaged areas of the embryo – the region localised anteriorly to the cephalic furrow shows lower levels of morphogenetic activity whilst the posterior region shows high levels of morphogenetic activity. The shown embryo is expressing *D*E-CadherinGFP. Scale bar - 50 µm. **B)** A FRAP experiment consists in 4 different phases of image acquisition characterised by different temporal resolution and duration: prebleach (1frame/5seconds; 25seconds); fast postbleach (1frame/1second; 60seconds); medium postbleach (1frame/5seconds; 600seconds); slow postbleach (1frame/30seconds; 600 seconds). Still images of a SpiderGFP FRAP experiment movie are shown as an example to highlight all phases. Scale bar - 10 µm. **C)** FRAP recovery curve of a *D*E-CadherinGFP experiment using a constant image acquisition rate of 5 seconds with corresponding closeup of the initial 100 seconds as shown in the boxed area located on the right. **D)** FRAP recovery curve of a *D*E-CadherinGFP experiment using our FRAP setup (see Fig.2B) with corresponding closeup of the initial 100 seconds as shown in the boxed area located on the right.

We conducted FRAP assays in two regions of stage 9 embryos, a posterior region, encompassing both the ventral and dorsal region of the germband, and an anterior region, localised anterior to the cephalic furrow (Figure 2A). The two regions differ in their ‘morphogenetic activity’, in that the posterior region shows dynamic remodelling of junctions due to cell intercalations, oriented cell division and cell elongation, while the anterior region is supposed to be less active at this stage, since the invagination of the stomodeum is initiated about an hour later [10]. Each FRAP experiment thus consisted of two sequentially acquired movies – one made in the anterior and one in the posterior region of the same embryo (Fig. 2A).

**Figure 3:**
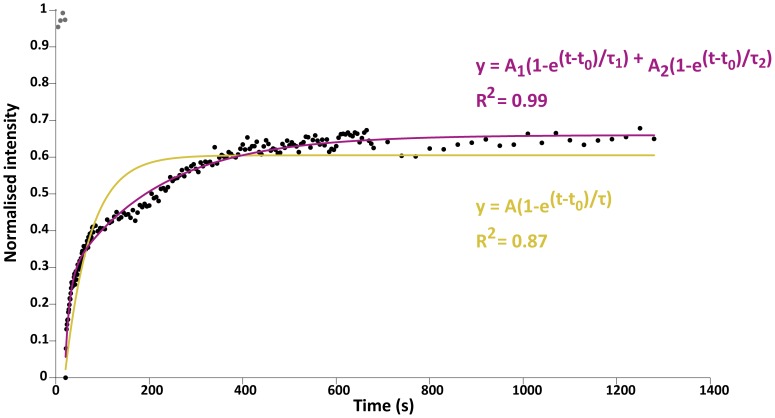
Double exponential fits better describe the FRAP data. FRAP recovery curve of a *D*E-CadherinGFP experiment with the newly developed imaging protocol with two different fitting curves and their parameters. Normalised raw data (black); single exponential fitting curve (yellow) and double exponential fitting curve (purple). The different curve fitting equations are shown as well as the fit correlation with the raw data (R^2^).

The experimental setup of our FRAP assay consisted in varying temporal imaging acquisition rates post-bleach, performed consecutively in the anterior and posterior region of the embryo (see Materials and Methods) (Fig. 2B). This allowed grasping at the same time the very brisk recovery immediately after bleach and the slower, more ample increase over longer times. A constant acquisition rate would miss either the fast response when using long time intervals, or the complete recovery in case of imaging short intervals only (Fig. 2C-D’). We observed that in all cases the recovery curves resembled the sum of two exponentials 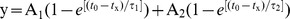 (Fig. 3). We retained this function as an empirical fitting model. Other *ad-hoc* models with less parameters, *e.g.* the single exponential, could not fit as well the experimental curves (see the fit correlation values (R^2^) in Fig. 3). The two-exponential model has the benefit of having a very limited number of free parameters and hence better describes the experimental recovery curves (Fig. 3). Moreover, the two exponential components each grasp separate time-scales: the early, quick recovery is described by the exponential with the smallest τ (τ_1_ here) and the later, slower recovery is described by the largest τ (τ_2_). This separation in the fitting process works, because the quick and slow responses have time-scales that differ by several orders of magnitude (see below). As suggested previously [35] we propose that the fast and slow recovery could be the result of diffusion of molecules from outside the region of interest (ROI) and of biosynthetic delivery of proteins via intracellular trafficking routes, respectively, though other alternatives, such as fast and slow membrane trafficking routes cannot be excluded.

Once all double exponential fitting curves were obtained from all FRAP experiments for all different markers and normalised (see Materials and Methods), we performed statistical analysis of all parameters. The time-scale parameters τ_1_ and τ_2_ gave us the recovery kinetics, whilst A_1_ and A_2_ measured the relative amount of fluorophore (mobile fraction) used in the quick and slow recovery, respectively. By following these four parameters, we could monitor changes in the recovery dynamics and changes in the pre-eminence of one versus another.

**Figure 4:**
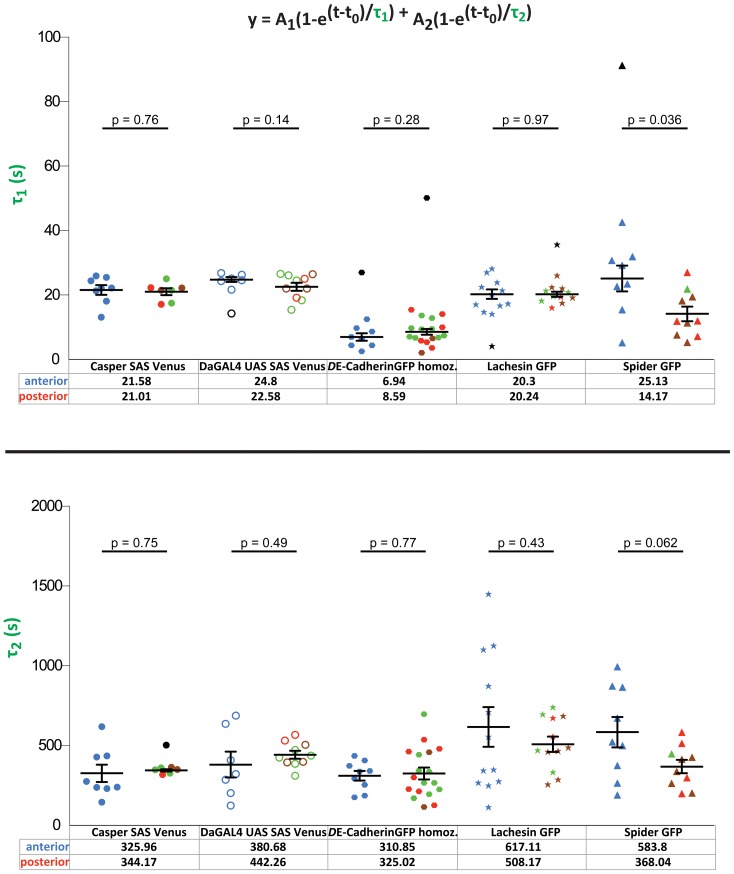
Kinetic values of all membrane markers in wild-type embryos. τ_1_ mean values and τ_2_ mean values with corresponding error bars (mean ± SEM) in both anterior and posterior regions of wild-type embryos. All membrane markers and their different conditions are shown. Each point refers to a different experiment. The values in the bottom refer to the mean value of the kinetic parameter assessed in both the anterior and posterior regions of the embryo. Note the difference in order of magnitude of the two kinetic parameters. Blue refers to movies performed in the anterior. Green refers to movies performed in the dorsal posterior, brown to movies in the ventral posterior, red to movies where it was not possible to establish whether they were dorsal or ventral and black to outliers identified by the MATLAB script. The significance values (p-values) between every condition are pointed out in the figure.

No differences in τ_1_ and τ_2_ of Sas-Venus, *D*E-CadherinGFP and LachesinGFP were observed between the anterior and the posterior region (Fig. 4,; blue symbols mark the anterior and red/green circles the posterior regions). However, a significant difference of SpiderGFP in τ_1_ recovery was observed between the two areas of the embryo, in that the fluorescence in the anterior recovered more slowly than in the posterior of the embryo (25.13s vs 14.17s) (Fig. 4, top, last two data sets). A similar behaviour was observed for τ_2_, as the recovery rate in the anterior was slower than that in the posterior of the embryo (583.8s vs. 368.04s) (Fig. 4, bottom, last two data sets). To summarise, only the general membrane marker SpiderGFP showed a difference in recovery, in that the recovery was slower in the anterior region in comparison to the posterior region. These differences reflect the varying levels of morphogenetic activity in the embryo – cells where no intercalation occurs (anterior) have longer recovery times after bleaching, whereas cells undergoing intercalation (posterior) are much faster in recovering the levels of fluorescence of the general membrane marker SpiderGFP.

**Figure 5:**
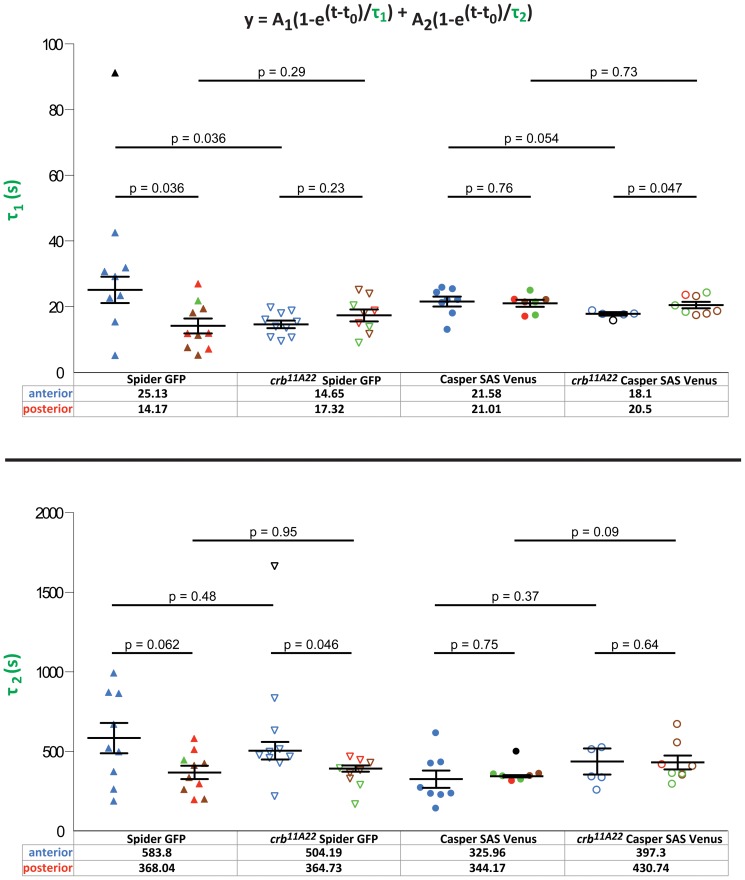
Kinetic values of SpiderGFP and Casper-Sas-Venus in *crb^11A22^* embryos. Kinetic values in *crb^11A22^* embryos expressing SpiderGFP and Casper-SAS-Venus. τ_1_ mean values and τ_2_ mean values with corresponding error bars (mean ± SEM) in both anterior and posterior regions of *crumbs^11A22^* embryos. All membrane markers and their different conditions are shown. Each point refers to a different experiment. The values in the bottom refer to the mean value of the kinetic parameter assessed in both the anterior and posterior regions of the embryo. Note the difference in order of magnitude of the two kinetic parameters. Blue refers to movies performed in the anterior. Green refers to movies performed in the dorsal posterior, brown to movies in the ventral posterior, red to movies where it was not possible to establish whether they were dorsal or ventral and black to outliers identified by the MATLAB script. The significance values (p-values) between every condition are pointed out in the figure.

To reveal a role of *crb* for trafficking of membrane proteins, we performed FRAP assays of SpiderGFP and Sas-Venus in *crb* mutant embryos. Again, we measured τ_1_ and τ_2_ in the anterior and posterior region. As described above, τ_1_ and τ_2_ of SpiderGFP had higher values in the anterior compared to the posterior in wild-type embryos (25.13s vs. 14.17s and 583.8s vs. 368.04s)) (Fig. 5, left; blue symbols mark the anterior and red/green circles the posterior regions). In the absence of *crb*, the difference of τ_1_ was completely abolished and the recovery rates were similar in the anterior and posterior *crb* mutant embryos (14.65s vs. 17.32s) (Fig. 5, top, left). The difference of τ_2_ values between anterior and posterior observed in wild-type embryos was maintained in *crb* mutant embryos. τ_2_ showed higher values in the anterior compared to those in the posterior region (wild-type: 583.8s anterior vs. 368.04s posterior, and *crb*: 504.19s anterior vs. 364.73 s posterior) (Fig. 5, bottom, left). We recognised that the kinetic values of τ_1_ in the anterior of *crb* mutant embryos (14.65s) were decreased in comparison to wild-type (25.13s), while the posterior values remained similar (14.17s vs. 17.32s) Fig. 5, top, left). A similar observation was obtained for τ_2_, which was reduced in *crb* mutant embryos in comparison to wild-type only in the anterior region (583.8s in wild-type vs. 504.19s in *crb*), but not in the posterior (368.04s in wild-type vs. 364.73s in *crb*). To summarise, absence of *crb* affected the kinetics of a general membrane marker, SpiderGFP, only in the anterior region of the embryo. SpiderGFP recovered faster than in wild-type, so that the values measured in the anterior were more similar to that in the posterior region, thus abolishing the difference between anterior and posterior observed in wild-type embryos.

We next analysed the consequences of the loss of *crb* on the behaviour of the apical marker Sas-Venus. The only significant difference we observed was a decrease in the mean value of τ_1_ in the anterior region of the embryos (21.58s in wild-type vs. 18.1s in *crb*) (Fig. 5, top, right, blue symbols). No effect on the behaviour of Sas-Venus was observed in the posterior (21.01s in wild-type vs. 20.5s in *crb*). Equally, no significant differences in the τ_2_ values of wild-type and *crb* mutant embryos were observed in the anterior (325.96s vs. 397.3s) and the posterior regions (344.17s vs. 430.74s).

Taken together, our results show that the absence of *crb* enhances the diffusion/fast delivery (τ_1_) of both SpiderGFP as well as Sas-Venus, but only in the plasma membrane of the anterior region.

## Discussion

The FRAP experiments presented here aimed to analyse whether the differences observed in morphogenetic behaviour of epithelia in the head and the extending germband of the *Drosophila* embryo were reflected by the kinetics of membrane proteins. Strikingly, from the four proteins analysed, only SpiderGFP showed a significant difference between the anterior and the posterior. Cells in the anterior, where no intercalation occurs take longer to recover their fluorescence after the bleach, whereas cells undergoing intercalation (posterior) are much faster in recovering the levels of fluorescence. In wound-healing assays performed in *Drosophila* embryos, SpiderGFP showed the same behaviour as the pleckstrin homology domain of PLC (phospholipase Cγ) and GAP43 (growth-associated protein 43), despite the fact that all three are differently attached to the membrane. This suggested that all of them reflect the behaviour of the membrane in general [36].

The unique behaviour of SpiderGFP observed could be explained by the fact that it is the only one of the four proteins analysed that is linked to the membrane via palmitoylation. Therefore, the anterior-posterior difference in its dynamics cannot easily be explained by differences in protein/vesicle trafficking. They may rather hint to differences in trafficking and/or mobility within the membrane.

Unexpectedly, however, the higher morphogenetic activity in the germband due to convergent extension movements is not reflected by a higher turnover of *D*E-Cadherin in this region compared to the anterior region, suggesting that the apico-basal boundary is maintained. This is different from the pupal wing epithelium, where the hexagonal packing of cells depends on polarised trafficking of *D*E-Cadherin during junction remodelling [37].

There is a prominent effect of loss of *crb* on τ_1_ of SpiderGFP and Sas-Venus, but only in the anterior region. Recovery is enhanced in the absence of *crb* and approximates its values to the ones measured in the germband. This suggests that *crb* plays an important role on membrane dynamics particularly in the procephalic region. Common to both proteins is their association with the apical membrane. This suggests that Crb affects specifically apical proteins, independent of the way they are associated with the membrane, a result that is in agreement with the apical localisation of Crb itself. So far, we can only speculate about the mechanism by which Crb influences the dynamics of SpiderGFP and Sas-Venus. Crb could stabilise the underlying membrane-associated cytoskeleton, and/or it may modify the characteristic features of the membrane. Both mechanisms could act on transmembrane (Sas) and palmitoylated proteins (Spider). Support of the former model comes from the observation that loss of Crb results in loss of β_H_ spectrin [25],[38]. Loss of β_H_ spectrin, in turn, could lead to a destabilisation of the membrane-associated cytoskeleton and enhanced protein turnover. In fact, a higher rate of endocytosis upon reduction of spectrin was described previously [39],[40], but this is more likely to act on the slow phase of recovery. Alternatively, the effect of *crb* on the fast phase of recovery could be explained by a faster diffusion in the membrane, which could be due to the deterioration of a diffusion barrier within the membrane due to the loss of the Crb complex. It is well established that the highly complex organisation of the plasma membrane itself has an impact on the diffusion, stability and trafficking of proteins [41],[42]. It is tempting to speculate that Crb may, directly or indirectly, modify membrane characteristics, which would result in a faster recovery of membrane proteins by lateral diffusion.

Unexpectedly, lack of *crb* affects the dynamics in the anterior region, rather than in the germband. In fact, the head epidermis falls apart earlier in *crb* mutant embryos than the epidermis in the trunk [43], although no major defects in epithelial integrity were observed in the anterior region at early stages of development (data not shown). Nevertheless, the anterior region is subject to morphogenetic changes due to postblastodermal divisions [44] and delamination of neuroblasts, the precursors of the nervous system [45],[46],[47]. Both processes occur earlier in the head than in the trunk and may require additional mechanisms ensuring tissue stability. Therefore we suggest, that the faster recovery of SpiderGFP and Sas-Venus observed in *crb* mutant embryos in the procephalic region are the first signs of tissue instability. The data presented here reveal a novel function of *crb* in epithelial morphogenesis by influencing the dynamics of membrane proteins.

## Materials And Methods

### Cloning Of Sas-Venus And Establishment Of Transgenic Lines

Stranded at Second (Sas) CDS was obtained from the *Drosophila* Genomics Resource Center (LD44801). The low complexity region of Sas replaced by Venus consisted in 461bp as determined by the restriction sites SpeI and XhoI. Venus was placed between two linker sequences (GGSGGGGSGG) in order to optimise its solubility and folding within SAS. SAS-Venus was cloned into pCasper4 with a tubulin promoter (gift from Suzanne Eaton lab), tub-SAS-Venus, and into pUAST, giving rise to UAS-SAS-Venus.

P-element transformation of the constructs was done according to the procedure described by [48]. We used *w^1118^* as recipient strains. Several independent transgenic lines were established. Correct localisation of transgene-encoded Venus-tagged Sas protein in embryos was confirmed by comparing Venus fluorescence with antibody stainings against endogenous Sas protein, using anti-Sas (dilution 1:500; kindly provided by D. Cavener), and anti-*D*E-cadherin antibody to mark the *zonula adherens* (dilution 1:50 [49]) and standard fixation protocols of *Drosophila* embryos [50].

### *Drosophila* Stocks

Flies were raised on conventional cornmeal agar at 25°C. See fly list in Table 1.

**Table 1:** Fly List.

Fly line	Description
*D*E-CadherinGFP	*D*E-Cadherin fused with GFP under control of ubiquitin promoter on 2^nd^ chromosome; homozygous viable 52]; transmembrane protein
SpiderGFP	FlyTrap line: *gish* fused with GFP under endogenous promoter on 3^rd^ chromosome; homozygous viable 53]; palmitoylated protein
LachesinGFP	Protein trap line: *lachesin* fused with GFP under endogenous promoter on 2^nd^ chromosome; homozygous viable (kindly provided by the Klämbt Protein trap consortium); GPI-linked protein
Casper-Sas-Venus	*Stranded at Second* fused with Venus under tubulin promoter on 3^rd^ chromosome; homozygous viable (this study); transmembrane protein
UAS Sas-Venus	*Stranded at Second* fused with Venus under UAS control region on 3^rd^ chromosome; homozygous viable (this study); transmembrane protein
daGAL4	*daughterless*GAL4 - ubiquitous and strong driver line for expression of UAS constructs on 3^rd^ chromosome; homozygous viable 24,54]
*crb^11A22^* SpiderGFP/TTG	SpiderGFP recombined with *crb^11A22^* with TTG balancer (TM3, P{GAL4-twi.G}2.3, P{UAS-2xEGFP}AH2.3, Sb^1^ Ser^1^) (this study)
*crb^11A22^* Casper-SAS-Venus/TTG	pCasper SAS-Venus 1 recombined with *crb^11A22^* over TTG balancer (TM3, P{GAL4-twi.G}2.3, P{UAS-2xEGFP}AH2.3, Sb^1^ Ser^1^) (this study)

### Embryo Collections

Flies were placed in cages with apple juice agar plates containing yeast. After two hours, the plates were collected and left at 25°C for roughly 5h, giving rise to embryos at germband elongation (stage 8–10).

### Imaging Of Live Embryos By Laser Confocal Microscopy

Embryos were dechorionated in bleach for 2m45s, rinsed with water and placed on slides containing HaloCarbon Oil 700 (Sigma-Aldrich) with two coverslips (thickness 1,5; 22mm×22mm, Corning) on each side, creating an artificial chamber when covered with a coverslip (thickness 1; 24mm×50mm, Menzel-Glaeser).

FRAP experiments were conducted in an inverted microscope with motorised stage (Zeiss LSM 510 DuoScan, Carl Zeiss MicroImaging, Inc.), using the 488nm line of an Argon laser with a 505–530 emission filter for GFP and Venus and a 405nm laser diode for the bleaching. All images were captured with a C-Apochromat 1.2 NA 40× water immersion objective (Carl Zeiss MicroImaging, Inc.) with a zoom of 3 for the FRAP experiments. All images consisted in 2 µm optical slices.

### Photobleaching And Analysis

FRAP experiments were performed by photobleaching a circular ROI (region of interest) encompassing the target cell and its surrounding neighbours and then monitoring fluorescence recovery. Since FRAP experiments were performed in both the anterior and posterior regions of the same embryo, there is a time delay of roughly 22 minutes between the start of both movies (the duration of a FRAP experiment)..

To achieve the different temporal acquisition rates, a macro was created using VisualMacro Editor (Carl Zeiss MicroImaging, Inc.). Images were analysed with FIJI software [51]. To compensate for cell drift, the Linear Stack Alignment with SIFT plugin was used. Fluorescence value measurements were then exported to Microsoft Excel where they were normalised and scaled between 0–1 using:



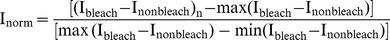



Subsequently, these Excel files containing the normalised values of fluorescence recovery were imported to a MATLAB script, which performed the curve fitting analysis, plotted these and then did a statistical analysis of the various parameters obtained from the double exponential equation used for the fit. Since the FRAP experiments were conducted in the same optical slice throughout the whole duration of the experiment, eventual shifts in the z-axis had to be manually curated and then compiled in a master file stating the timepoint at which an eventual z-axis shift had occurred. This was then used as a reference by the MATLAB script when using the curve fitting module.

Outliers in the fitted parameters were identified as follows – for each parameter distribution, q1 and q3, were calculated, which represent 25% and 75% percentile, respectively. Values larger than q3+1.5×(q3−q1) were deemed as outliers and not included in the mean, standard deviation and t-tests calculations.
